# CAR/FoxP3-engineered T regulatory cells target the CNS and suppress EAE upon intranasal delivery

**DOI:** 10.1186/1742-2094-9-112

**Published:** 2012-05-30

**Authors:** Moa Fransson, Elena Piras, Joachim Burman, Berith Nilsson, Magnus Essand, BinFeng Lu, Robert A Harris, Peetra U Magnusson, Eva Brittebo, Angelica SI Loskog

**Affiliations:** 1 Department of Immunology, Genetics and Pathology, Science for Life Laboratory, Uppsala University, Rudbeck Laboratory C11, Dag Hammarskjoldsv 20, SE-75185, Uppsala, Sweden; 2 Department of Pharmaceutical Biosciences, Uppsala University, BMC, Husarg 3, SE-75124, Uppsala, Sweden; 3 Department of Neuroscience, Uppsala University, Uppsala University Hospital Entr 70, SE-75185, Uppsala, Sweden; 4 Department of Immunology, University of Pittsburgh, 320 East North Avenue, Pittsburgh, PA, 15212, USA; 5 Applied Immunology, Department of Clinical Neurosciences, Karolinska Institutet, Center for Molecular Medicine, Karolinska Hospital at Solna, Solna, Sweden

**Keywords:** MS, redirected cells, T regulatory cells, EAE, FoxP3, Myelin oligodendrocyte glycoprotein (MOG)

## Abstract

**Background:**

Multiple sclerosis (MS) is an autoimmune disease of the central nervous system (CNS). In the murine experimental autoimmune encephalomyelitis (EAE) model of MS, T regulatory (Treg) cell therapy has proved to be beneficial, but generation of stable CNS-targeting Tregs needs further development. Here, we propose gene engineering to achieve CNS-targeting Tregs from naïve CD4 cells and demonstrate their efficacy in the EAE model.

**Methods:**

CD4^+^ T cells were modified utilizing a lentiviral vector system to express a chimeric antigen receptor (CAR) targeting myelin oligodendrocyte glycoprotein (MOG) *in trans* with the murine *FoxP3* gene that drives Treg differentiation. The cells were evaluated *in vitro* for suppressive capacity and in C57BL/6 mice to treat EAE. Cells were administered by intranasal (i.n.) cell delivery.

**Results:**

The engineered Tregs demonstrated suppressive capacity *in vitro* and could efficiently access various regions in the brain via i.n cell delivery. Clinical score 3 EAE mice were treated and the engineered Tregs suppressed ongoing encephalomyelitis as demonstrated by reduced disease symptoms as well as decreased IL-12 and IFNgamma mRNAs in brain tissue. Immunohistochemical markers for myelination (MBP) and reactive astrogliosis (GFAP) confirmed recovery in mice treated with engineered Tregs compared to controls. Symptom-free mice were rechallenged with a second EAE-inducing inoculum but remained healthy, demonstrating the sustained effect of engineered Tregs.

**Conclusion:**

CNS-targeting Tregs delivered i.n. localized to the CNS and efficiently suppressed ongoing inflammation leading to diminished disease symptoms.

## Background

Multiple sclerosis (MS) is an autoimmune disease of the central nervous system (CNS) involving autoreactive T cells recognizing myelin epitopes. Activated T cells invade the CNS, recruit peripheral mononuclear phagocytes and demyelination in the brain and spinal cord tissue, ultimately leading to impaired neuronal transmission [1]. T regulatory cells (Tregs) have the capacity to regulate ongoing immune reactions and are important in the control of autoimmunity [2,3]. Tregs exert their immunosuppressive functions via secretion of inhibitory cytokines, by interfering with the metabolism of T cells and/or in an undetermined contact-dependent manner. Furthermore, Tregs block T cell activation indirectly via their interaction with antigen-presenting cells (APCs), preventing APC maturation and consequently downregulate their expression of costimulatory molecules and cytokine secretion [4,5]. Many studies have investigated the role of Tregs and their suppressive function in MS patients and despite some contradicting results, likely due to the multiple definitions of Treg subclasses, it has been concluded that their suppressive activities are impaired during disease progression [6-8]. MS patients may therefore benefit from Treg cell therapy to restore this insufficient immunosuppressive capacity.

It has also been demonstrated that Tregs play a critical role in the protection and recovery of the animal model of MS, experimental autoimmune encephalomyelitis (EAE). Depletion of Tregs inhibits natural recovery from EAE whereas transfer of Tregs to recipient mice reduces disease severity [9,10]. Transfer of antigen-specific Tregs derived from TCR transgenic mice was more effective than polyclonal Tregs in controlling murine models of both autoimmune gastritis [11] and MS [12]. However, adequate numbers of antigen-specific Tregs are difficult to achieve for adoptive transfer. A chimeric antigen receptor (CAR) was used to redirect Tregs to a desired antigen in a colitis model [13,14]. Cultured Tregs may change their suppressive phenotype posttreatment, and this might be detrimental for patients. For example, Xu and coworkers have reported that Tregs in the absence of TGFβ can differentiate into Th17 cells, which are considered an integral cause of autoimmune manifestations in MS [15]. Since FoxP3 is fundamental for the differentiation and maintenance of Tregs, a stable expression of FoxP3 by genetic engineering may block Treg conversion into effector cells and thereby provide a safer option for patients. In a model of arthritis, FoxP3 was coexpressed with an antigen-specific TCR to achieve multiple stable targeting Tregs [16]. By co-expressing FoxP3 with a chimeric antigen receptor (CAR) [17] targeting myelin oligodendrocyte glycoprotein (MOG) in naive CD4^+^ T cells we can generate sufficient numbers of stable Tregs that localize to the CNS. The role of the CARαMOG receptor is to attach the Treg to the vicinity of MOG + oligodendrocytes to prevent immune attacks against these cells.

In the present study, engineered Tregs were analyzed for their suppressive function, capacity to localize to the CNS upon intranasal (i.n) or intraperitoneal (i.p) cell delivery, and for their therapeutic capacity in EAE symptomatic mice.

## Materials and methods

### Antibody production, purification and immunohistochemistry

Hybridoma cell line 8–18 C5 [18] was cultured in RPMI 1640 medium supplemented with 10% fetal calf serum. Antibodies were purified using protein A affinity chromatography (HiTrap MabSelect, GE Healthcare, Little Chalfont, UK) following addition of 0.5 M trisodium citrate (Sigma-Aldrich Corp., St Louis, MO, USA) to the clarified supernatant. The column was washed with 500 mM sodium citrate pH 8.5 and the antibody fraction was eluted with 0.1 M glycine (Sigma-Aldrich) at pH 2.7. The eluate was neutralized using Tris-HCl (Sigma) at pH 8 and concentrated using a JumboSep ultrafiltration device and 10kD cutoff filter (Pall Gellman, WWR International, Stockholm). Specificity of the antibody was confirmed through Western blotting analyses of whole mouse myelin and recombinant MOG.

For immunohistochemical MOG detection sections from a naïve mouse brain were fixed in 4% paraformaldehyde and rinsed with PBS-Tween before addition of peroxidase blocking reagent (EnVision, DakoCytomation, Glostrup, Denmark). Sections were first blocked using 100 μl (1:100) Fc-receptor block (Serotec, Kidlington, UK) and thereafter staining was performed with 200 μL (1:50) αMOG antibodies (8.18 C5, C. Linington) followed by 200 μl (1:50) rabbit-α-mouse Ig (EnVision, DakoCytomation) for 30 minutes each. AEC solution (DakoCytomation) was applied for colour development and sections were finally counterstained with hematoxylin prior to microscopic analysis.

### Chimeric antigen receptor (CAR) construct

**Figure 1:**
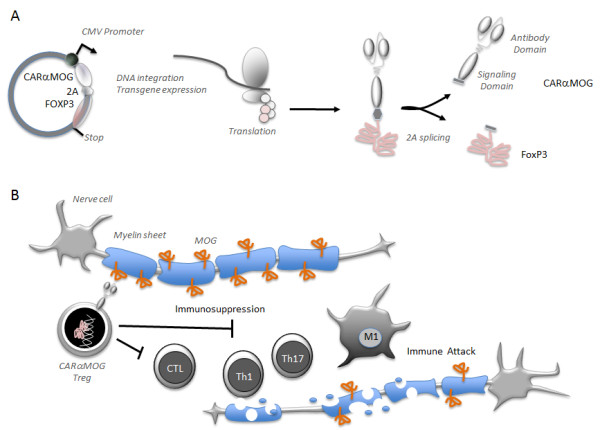
**CNS-targeting receptor and FoxP3 translation and function. (A)** The CARαMOG-FoxP3 vector contains a scFv cloned from the 8.18 C5 hybridoma. The scFv is linked via an antibody hinge region to the transmembrane and intracellular part of a CD3 zeta chain. The zeta chain is further fused to an intracellular CD28 domain. The murine FoxP3 gene was inserted into the construct after a 2A peptide sequence. Upon translation, the whole expression cassette is translated into a CARFoxP3 fusion protein that is self-cleaved at the 2A site to produce the two separate proteins CARαMOG and FoxP3. **(B)** CARαMOG and FoxP3 is transported to the cell surface and nucleus, respectively. At the cell surface CARαMOG can bind to MOG + cells to attach the Treg to those cells and prevent immune attacks on MOG + cells such as oligodendrocytes in the CNS. FoxP3 will drive the Treg phenotype by regulating gene transcription in the nucleus.

The CARαMOG-FoxP3 vector (Figure 1A) was constructed as follows: a single chain variable fragment (scFv) was cloned from hybridoma (8.18 C5) producing anti-rat myelin oligodendrocyte glycoprotein (MOG) antibodies. The scFv was linked via an antibody *hinge* region to the transmembrane and intracellular part of a *CD3ζ* chain, which was in turn fused to an intracellular *CD28* domain. The murine *FoxP3* gene was inserted into the construct and separated from the CAR gene by a 2A peptide (described in reference [19]). The final CARαMOG-FoxP3 construct was inserted into the lentivector pRRL-CMV (kind gift from R Houeben, Leiden University Medical Center, Netherlands). Lentiviruses (Lenti-CARαMOG-Foxp3 and Lenti-Mock, Lenti-GFP) were produced by co-transfecting 293FT cells with pLP1, pLP2 and pLP/VSVG (Invitrogen, Paisley, UK). Virus supernatants were harvested on days 2 and 3 and concentrated by ultracentrifugation. The amino acid sequence for the CARαMOG receptor is given in Additional file 1: Figure S1.

### Genetic engineering of T cells

Murine naive CD4 cells were sorted using the MACS bead system (Miltenyi, Bergisch, Germany) and prestimulated with an initial dose of 1 μg of both anti-CD3 and anti-CD28 immobilized antibodies (BD Biosciences, San Diego, CA, USA) as well as IL-2 (R&D systems Inc., Minneapolis, MN, USA) for three days prior to viral transduction, since a good viral gene transduction of T cells require cycling cells. 50 μl of viral supernatants was added to 5 × 10^5^ stimulated CD4^+^ T cells in 100 μl RPMI-1640 medium supplemented with 1% sodium pyruvate, 1% nonessential amino acids, 10% fetal bovine serum, 1% penicillin/streptomycin (all from Invitrogen, Paisley Scotland) and 8 μg/ml Polybrene (Sigma-Aldrich Corp., Saint Louis, MO, USA). Cells were incubated for four hours at 37 °C, 5% CO_2_ followed by addition of 300 μl of media (as above) supplemented with 100U IL-2. The following day, media (as above) was replaced with fresh media supplemented with 80U IL-2. Cells were cultured for seven days with addition of 80U of IL-2 every second day. Transduction efficiency was analyzed three-to-six days post-transduction. Transduced cells were incubated for 10 minutes at 4 °C with a FITC-conjugated mAb specific for the IgG-kappa in the scFv (BD Biosciences, San Diego, CA, USA), washed with PBS and resuspended in 1% paraformaldehyde (PFA) in PBS. Samples were analyzed for surface expression of CAR or intracellular green fluorescent protein (GFP) expression using a FACScanton (BD Biosciences, San Diego, CA, USA).

### EAE induction and Treg cell administration

Female C57BL/6 mice were purchased from Taconic, Lille Skensved, Denmark. Mice were housed in the Department of Animal Resources facilities at Uppsala University and used at five to eight weeks of age. Studies were approved by the regional animal ethics committee in Uppsala (C28/10). EAE was induced by subcutaneous (s.c.) immunization in both hind and front limbs with 200 μg MOG_35-55_ peptide emulsified in complete Freunds’ adjuvant (CFA) (Difco Laboratories, Detroit, MI, USA) containing 5 mg/ml *Mycobacterium tuberculosis*. *Pertussis* toxin (100 ng i.p) (Sigma-Aldrich Corp., Saint Louis, MO, US) was given at the time of immunization and a second dose two days later. Disease severity was monitored according to the following scale: 0, no disease; 1, flaccid tail; 2, hind limb weakness; 3, hind limb paralysis; 4, fore limb weakness; 5, moribund. When the mean score value was 3 (usually at day 15), mice were treated using cell therapy. Cells (1 × 10^5^ CAR or Mock-transduced Tregs diluted in 10uL PBS) or phosphate-buffered saline (PBS) were administered i.n. in 5μL PBS using a plastic catheter connected to a pipette (polyethylene tube, Becton Dickinson, Franklin Lakes, NJ, USA) inserted for 3 mm in both nasal nostrils during anesthesia (0.05 to 0.1 mg ketalamine-xylazine mixture/10 g body weight; ketamine 50 mg/ml, Pfizer AB, Sollentuna, Sweden; xylazine 20 mg/ml, Bayer AG Animal Health, Business Group, Leverkusen, Germany). For i.p. cell therapy 1x10^5^ cells (CAR or Mock-transduced Tregs) diluted in 100 μL PBS were injected. Mice were sacrificed with gaseous CO_2_ and brains were excised and fixed either in ice-cold 4% phosphate-buffered formaldehyde (pH7.4) or isopentane with dry-ice for paraffin-embedding or frozen- sectioning, respectively. Tissues embedded in low-melting paraffin after grade scale alcohol dehydration and xylene treatment were sectioned in the sagittal plane (4 μm) through the brain, mounted on gelatine-coated glass and used for immunohistochemistry.

### Tissue localization of engineered Treg cells in naïve mice

1 × 10^4^ GFP/CARαMOG-FoxP3-engineered CD4^+^ T cells diluted in 5 μl PBS, Mock-transduced Tregs or PBS were administered i.n in the right nostrils of naïve animals as described above. Horizontal cryosections of the brain, (10 μm) were air-dried and kept at −80 °C. Tissue sections at the same level were selected following a quick staining with toluidine blue. Sections washed in cold PBS were quenched with 0.3% H_2_O_2_ in methanol, blocked with 2.5% normal horse serum for one hour followed by staining with anti-GFP primary antibody (1:300) ab390 (Abcam, Cambridge, UK) overnight at 4 °C. Thereafter, Alexa Fluor 488 anti-rabbit (1:200) secondary antibody was applied. Specificity controls for immunostaining included sections stained in the absence of primary antibody and staining of sections from a vehicle-treated mouse not receiving GFP^+^ cells. For detection of DNA/nuclei sections were overlain with Vectashield Mounting Medium containing 4′,6′-diamidino-2-phenylindoledihydrochloride(DAPI) (Vector Laboratories, Burlingame, CA, USA). Immunofluorescence images were captured using a Leica DMRBE fluorescence microscope, a digital camera (Nikon Dxm 1200 F Nikon Corp., Tokyo, Japan) and Nikon ACT-1 version 2.62 software. All images were processed in Adobe Photoshop and Illustrator CS4, and green (GFP) and blue (DAPI) channel images were merged using Photoshop software.

### Immunohistochemistry for nerve damage and repair

For myelin basic protein (MBP) and glial fibrillary acidic protein (GFAP) detection the avidin biotin complex (ABC) method and 3,3′ diaminobenzidine (DAB) as chromogen were used. Deparaffinized and rehydrated sagittal sections were rinsed with PBS and PBS-T. For GFAP antigen, demasking was performed in a microwave using 10 mM sodium citrate buffer. Endogenous peroxidase activity was blocked with 1 to 3% H_2_O_2_ in PBS-T and nonspecific background staining was blocked with 4% BSA in PBS. Sections were incubated overnight with the primary antibodies (MBP 1:200 Abcam, Cambridge, UK; GFAP 1:400 Millipore, Billerica, MA. USA). After washing, the sections were incubated with a biotinylated secondary antibody and then with ABC complex (both from Vector Laboratories, Burlingame, CA, USA). Immunoreactions were visualized with DAB (Sigma-Aldrich Corp., St.Louis, MO, USA). Sections were counterstained with hematoxylin. Finally, the tissue sections were rinsed gradually through a graded alcohol series and finally in xylene, and mounted immediately after with Pertex (Histolab, Göteborg, Sweden). The tissue sections were analyzed using an Olympus microscope (Olympus, Tokyo, Japan) and images were captured using a digital camera as described above. Results were analyzed in a blinded mode scoring the level of staining as weak, moderate or strong. Digital images were collected at the same time using identical settings with respect to image exposure time and image compensation setting. Images were processed using Adobe Photoshop and Illustrator CS4.

### Treg suppression assay

For *in vitro* suppression assays, 3 × 10^4^ CARαMOG-FoxP3 or Mock-transduced CD4^+^ T cells irradiated at 25 Gy were mixed in different ratios with αCD3/IL-2-stimulated splenocytes derived from a naïve healthy mouse in a total volume of 200 μl/well. The RPMI-1640 medium was supplemented with 0.1% sodium pyruvate, 1% nonessential amino acids, 1% Hepes buffer, 1% β-mercaptoethanol, 10% fetal bovine serum and 1% penicillin/streptomycin (all from Invitrogen, Paisley, UK). Cells were seeded in 96-well rounded-bottom tissue culture treated plates (Sarstedt, Newton, NC, USA) and incubated for 48 hours, after which 1 μCi of ^3^ H-thymidine (PerkinElmer, Waltham, MA, USA) was added per well. Cells were incubated for an additional eight hours before harvest to filters. The incorporated ^3^ H-thymidine was measured using a β-counter (Perkin Elmer Life Science, Turku, Finland). In some experiments, murine 2.5 × 10^4^ macrophages or 2.5 × 10^4^ MOG^+^ cells were added to cultures with CARαMOG-FoxP3-transduced CD4^+^ T cells in a 1:1 ratio. Activated macrophages were obtained via plastic adherence of splenocytes. Monocytes were activated by 1 μg lipopolysaccharide (LPS) and matured during one week in RPMI-1640 medium supplemented with 1% sodium pyruvate, 1% nonessential amino acids, 10% fetal bovine serum and 1% penicillin/streptomycin. MOG^+^ cells were generated via lentiviral gene transfer of murine MOG to 293 T cells. MOG expression was confirmed by histochemistry using αMOG antibodies (clone 8.18 C5) as described above.

### Quantitative PCR

Brain biopsies from EAE mice treated i.n. with CAR, Mock-transduced Tregs or PBS, respectively, were treated with tissue lysis buffer ATL (Qiagen, Hilden, Germany) at 60 °C for three hours followed by DNA purification using High Pure Viral Nucleic Acid kit (Roche, Basel Switzerland). cDNA was obtained using the Superscript II Reverse Transcriptase kit (Invitrogen, Paisley, UK). Quantitative-PCR was performed using the real-time system (iCycler, Bio-Rad Laboratories Inc., Hercules, CA, USA). The reaction was performed with SYBR green mix (BioRad). Primer pairs for β-actin were designed as follows: forward 5′-TTCCTTCCCAGAGTTCTTCCAC, reverse 5′-CCAGGATGGCCCATCGGATAAG (Cybergene AB, Huddinge, Sweden). Primers to detect IL-12 and IFNγ were designed as described previously [19,20]. In order to correct for variable amounts of DNA content between samples, all copy numbers were corrected to β-s. The mRNA copy number in 2 μL cDNA was evaluated in the experiments.

### Statistics

Significant differences between groups were calculated using GraphPad Software (La Jolla, CA, USA). The method for each individual calculation is stated in the Figure Legends. *P < 0.05, **P < 0.01, ***P < 0.001.

## Results

### Phenotype and function of engineered Tregs

**Figure 2:**
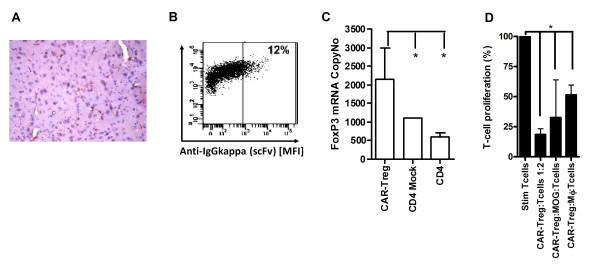
**Engineered Tregs express CAR and FoxP3. (A)** Antibodies from the 8.18 C5 hybridoma cross-reacted with murine myelin as demonstrated by immunohistochemistry (red colour expression). **(B)** CD4^+^T cells transduced with CARαMOG-FoxP3 lentivectors (CAR Tregs) were stained with fluorescein isothiocyanate (FITC)-conjugated antibodies and analyzed for surface expression of the scFv by flow cytometry. **(C)** FoxP3 copy number in transduced cells analyzed by quantitative PCR analysis. Expression of FoxP3 is significantly higher than both CD4-mock and naïve CD4^+^T cells (*P* < 0,05) as analyzed by Mann-Whitney using GraphPad prism software. The expression experiments were repeated for every cell generation with similar result. **(D)** CAR Tregs were mixed with αCD3/IL-2-stimulated T cells at a1:2 ratio and analyzed for suppressive ability in a thymidine-based assay. CAR Tregs were able to suppress activated T-cells (*P* < 0.05). CAR Tregs suppressed αCD3/IL-2-stimulated T cells in the presence of activated macrophages (*P* < 0.05) or MOG-expressing cells (*P* < 0.05). The experiments were repeated and statistical differences analyzed by Mann-Whitney test using GraphPad prism software.

Antibodies produced and purified from the 8.18 C5 hybridoma were tested for cross-reactivity to murine MOG. The selected MOG antibody detected murine MOG in the brains of naive mice (Figure 2A). A scFv from the 8.18 C5 hybridoma was generated and cloned into a murine CAR receptor and was then inserted in tandem with murine *FoxP3* into a lentiviral system to produce CARαMOG-FoxP3 viruses. Sorted and pre-activated naive CD4^+^ T cells were successfully transduced with the CARαMOG-FoxP3 lentivectors (CAR Tregs). Prior to stimulation and transduction CD4+ T cells are sorted from splenocytes. Transduced and expanded cells remain CD4 positive. Post-gene transfer the scFv of the CARαMOG receptor could be detected on the cell surface of approximately 10 to 15% of cells (Figure 2B) and the FoxP3 mRNA levels in the engineered cells were two-fold greater than that of Mock-transduced T cells (CD4 Mock) which include a population of naturally occurring Tregs (Figure 2C). Natural Tregs suppress activated T cells in a non-TCR restricted manner by contact dependent and independent mechanisms. CAR Tregs suppressed polyclonally stimulated T cells (*P* < 0.05) at a 1:2 ratio (Figure 2D) demonstrating the gained suppressive function. The role of the CARαMOG receptor is to attach the Treg to the vicinity of MOG + oligodendrocytes to prevent immune attacks against these cells (Figure 1B). To investigate that the CAR Tregs retained their suppressive function upon binding to MOG + cells, the suppressive function of polyclonally stimulated T cells were analyzed in cocultures with MOG + cells. In Figure 2D it is demonstrated that the CAR Tregs continue to suppress T cell proliferation in the presence of MOG + cells. Further, activated murine macrophages may produce cytokines or other factors that block the function of the CAR Tregs. Activated macrophages are part of the MS pathology and therefore CAR Tregs were cultured with such cells to determine if CAR Tregs were still suppressive against T cells. In our assay CAR Tregs were still able to suppress effector T cell proliferation in the presence of activated macrophages (Figure 2D; *P* < 0.05).

### *In vivo* localization of Tregs to the brain

**Figure 3:**
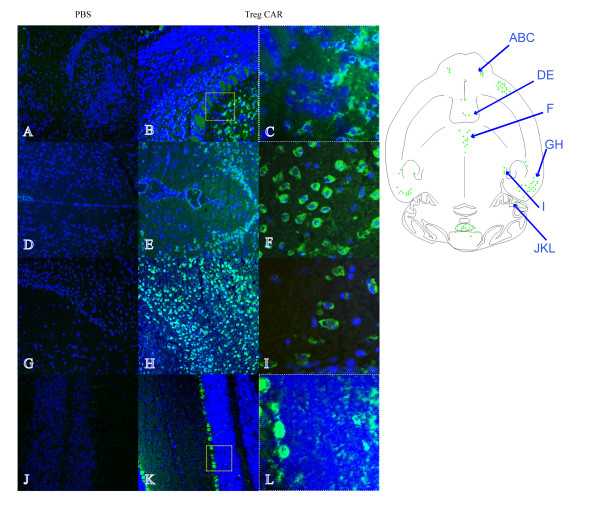
**Engineered Tregs localize to the CNS when administered intranasally in naive mice.** Treg cells were administered intranasally in the right nostril and the distribution of green fluorescent protein (GFP) in horizontal cryosections of the brain of naïve mice was studied 24 hours after the delivery. The schematic drawing describes a selective immunofluorescence in various brain regions (green spots). GFP immunofluorescence is present in the granular and to a lower extent in the external plexiform layer of the olfactory bulb **(B, C)**, lateral septal nucleus **(E)**, central medial thalamic nucleus **(F)**, ectorhinal cortex **(H)**, medial genic nucleus **(I)** and cerebellum **(K, L)** of a CAR Treg-treated naïve mouse. Corresponding areas showing no GFP fluorescence in a PBS-treated naïve mouse are **(A, D, G, J)**. Enlargements of areas in olfactory bulb and cerebellum (as indicated by boxes) are depicted in C and L. Detail of GFP immunofluorescence in the central medial thalamic nucleus and medial genic nucleus (F, I). Cell nuclei (blue) are stained with DAPI Original magnification 10× (A, B, D, E, G, H, J, K) and 40× (C, F, I, L).

CAR Tregs co-expressing GFP and CARαMOG-FoxP3 were used to evaluate *in vivo* targeting upon i.n. cell delivery in naïve mice. The overall localization of GFP immunofluorescence is illustrated in the schematic drawing in Figure 3. The green fluorescence was mainly localized in clusters of cells in the granular layer and the external plexiform layer of the olfactory bulb (Figure 3B, C), in the lateral septal nucleus (Figure 3E), in the central medial thalamic nucleus (Figure 3F), in the ectorhinal cortex (Figure 3H), in the medial genic nucleus (Figure 3I) and in the Purkinje cell layer and white matter of the cerebellum (Figure 3K, L). In addition, green immunofluorescence was observed in anterior olfactory nucleus and anterior orbital cortex (data not included). The green immunofluorescence was only observed in the soma and was preferentially present in the perinuclear part (Figure 3F, I). Although a unilateral dose of cells was given, the localization of immunofluorescence occurred both on the ipsilateral and contralateral sides of the brain. In the vehicle control animal no, or extremely weak, green background immunofluorescence could be detected (Figure 3A, D, G, J). The localization of immunofluorescence is summarized in Table 1.

**Table 1:** **Summary of GFP + cell distribution in mouse naive brain**^**a**^**.**

**Tissue**	**GFP Immunohistochemistry**
*Olfactory bulb*	
-granular layer	+++
-external plexiform layer	+
-periglomerular cells	-
Anterior olfactory nucleus	++
Anterior orbital cortex	++
Lateral septal nucleus	+++
Ectorhinal cortex	+++
Central medial thalamic nucleus	+
Subgeniculates nucleus	++
Lateral globus pallidus	-
*Cerebellum*	
-molecular layer	-
-purkinje cells	++
-white matter	+

^a^+++ intense staining, ++ moderate staining, + weak staining, - no staining.

### CNS-targeting CAR Tregs suppress active EAE

**Figure 4:**
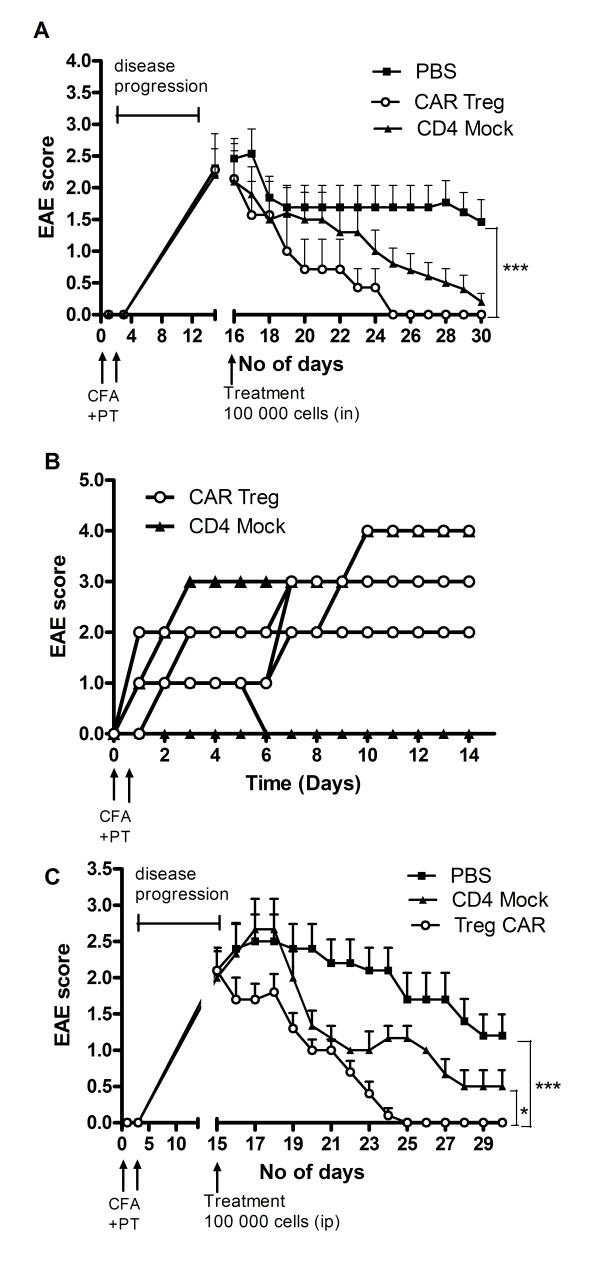
**CNS-targeting Tregs can reduce EAE symptoms.** (**A**) Ten mice in three groups were given 1 × 10^5^ CAR Tregs, CD4^+^ Mock T cells or PBS alone by i.n. administration at the peak of EAE inflammation (15 days post-EAE immunization) and thereafter were monitored for EAE symptoms. Ten days post cell treatment all EAE mice in the CAR Treg group were cured (*P* < 0.001). At end point (15 days post cell treatment) four out of ten EAE mice in the Mock-treated group still exhibited EAE symptoms. The experiment was repeated three times with similar results. **(B)** Symptom-free mice from each treatment group were given a second dose of EAE-inducing inoculum and monitored for EAE symptoms. CAR Treg-treated mice were able to resist EAE inflammation to a higher extent than CD4+ Mock-treated mice (*P* < 0.001). Pooled data from six EAE mice (three/group from two separate experiments) are shown in the figure. Scores for individual mice are shown separately in the figure. **(C)** Ten EAE mice in three groups were administered 1x10^5^ CAR Tregs, CD4^+^ Mock T cells or PBS alone by i.p. injection at peak of EAE inflammation (day 15). At end point (day 15 post-treatment) all mice in the CAR Τreg group were cured but six out of ten mice in the Mock CD4^+^ T cell group still exhibited EAE symptoms. Statistics are analyzed with Mann-Whitney test using GraphPad prism software. *P < 0.05, **P < 0.01, ***P < 0.001.

At the peak of EAE inflammation 1 × 10^5^ cells of each CAR Tregs and Mock CD4^+^ T cells, or PBS alone, was administered i.n. or i.p. to 10 mice per group. EAE mice responded well to cell therapy independently of administration route (Figure 4A to C). The EAE scores were initially reduced upon i.n delivery of either CAR Tregs or Mock CD4^+^ T cells. Seven days post-treatment only the CAR Treg treatment group exhibited a continuous reduction of clinical disease symptoms and at day 25 all mice (n = 10) were symptom-free (Figure 4A). At the same time point, only a few mice in the Mock control group (n = 5) that contained a normal rate of natural Tregs (see Figure 2C) were classified as being healthy. The remaining mice exhibited symptoms corresponding to a clinical score of 3. At day 30 also, the Mock control group had a good performance score and significantly differed from PBS controls (*P* < 0.05). At this time point healthy mice from each group, except the PBS treatment that did not cure mice, were re-challenged with an additional EAE-inducing inoculum using CFA and *pertussis* toxin. In the CD4^+^ Mock group all mice developed EAE symptoms by day two. In the CAR Treg group only one mouse developed weak EAE (score 1) (Figure 4B).

**Figure 5:**
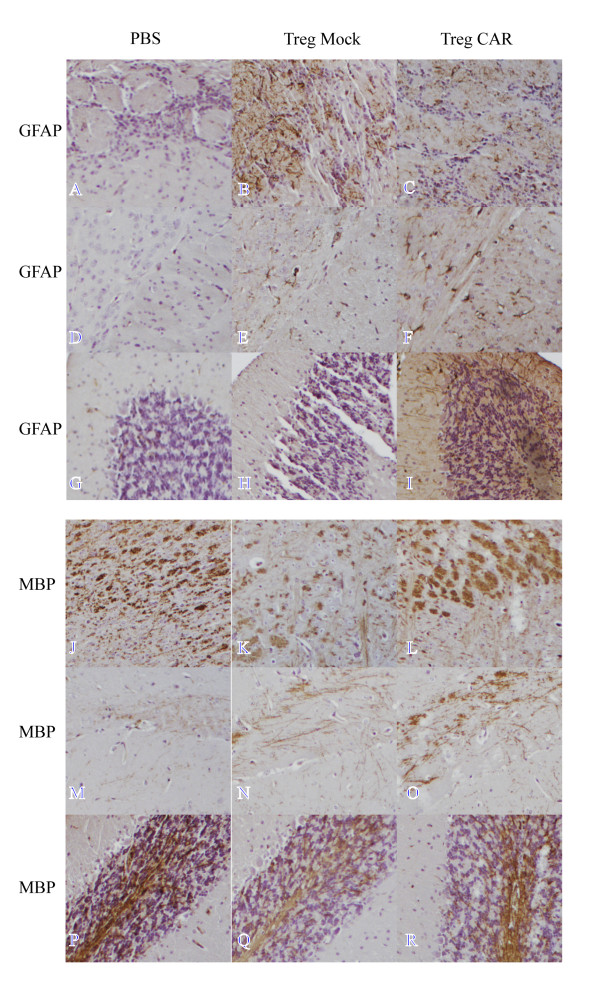
**Astrogliosis and remyelination after intranasal administration of CNS-targeting Treg.** Mice in three groups were given 1 × 10^5^ CAR Tregs, CD4^+^ Mock T cells or PBS alone by intranasal administration at the peak of EAE inflammation (15 days post-EAE immunization). Fifteen days post cell treatment the mice were killed and brain sections from each group (CAR Tregs, Mock CD4^+^ T cell and PBS) were analyzed for reactive astroglisosis using glial acidic fibrillary protein (GFAP) (**A**-**I**) and myelination using myelin basic protein (MBP) (**J**-**R**). GFAP was evaluated in olfactory bulb (**A**-**C**), corpus callosum (**D**-**F**), and cerebellum (**G**-**I**) of brain sagittal section from PBS, Mock CD4^+^ T cell and CAR Τreg-treated EAE mice. In the olfactory bulb of mice treated with Mock CD4^+^ T cells there was a strong staining for GFAP whereas this area in PBS-treated mice (A) and of CAR Treg-treated mice (C) exhibited a weak and moderate staining, respectively. There is an extremely weak staining in PBS-treated EAE mice, moderate staining in Mock CD4^+^ T cell-treated EAE mice and strong staining in CAR Treg-treated EAE mice for corpus callosum and cerebellum. MBP was evaluated in brain stem (**J**-**L**), hippocampus (**M**-**O**) and cerebellum (**P**-**R**) of brain sagittal sections in PBS, Mock CD4^+^ T cell and CAR Treg-treated EAE mice. In the brain stem and cerebellum of mice treated with CAR Tregs, there was a moderate staining for MBP (L, R) whereas the brain stem of Mock-treated mice (K,Q) and PBS-treated mice (J,P) exhibited a weak and strong staining, respectively. There is an extremely weak staining in PBS-treated EAE mice, a moderate staining in Mock CD4^+^ T cell-treated EAE mice and strong staining in CAR Treg-treated EAE mice for hippocampus. Original magnification 10×.

Upon examination of immunohistochemical markers for myelination (MBP) and reactive astrogliosis (GFAP), mice in the CAR Treg group exhibited confirmed recovery 15 days post-treatment (Figure 5). Reactive astrogliosis was evaluated in the olfactory bulb (Figure 5A-C), corpus callosum (Figure 5D-F), cerebellum (Figure 5G-I) and hippocampus (data not included). An increased GFAP staining was detected in both CAR Treg- and CD4^+^ Mock-treated EAE mice as compared to PBS-treated EAE mice. The level of staining was higher in CAR Treg-treated EAE mice as compared to CD4^+^ Mock-treated EAE in all areas except the olfactory bulb, where the level of staining was lower (Figure 5B, C). Myelination was evaluated in the brain stem (Figure 5J-L), hippocampus (Figure 5M - O) and cerebellum (Figure 5P-R), corpus callosum and olfactory bulb (data not included). The degree of myelination, as indicated by MBP staining in PBS-treated EAE mice compared to CAR Treg-treated mice in brain stem and in cerebellum, was slightly stronger (Figure 5J and P), whereas staining was weak or absent in the others areas. MBP staining in the brain of CD4^+^ Mock-treated EAE mice was lower compared to that in CAR Treg-treated mice.

**Figure 6:**
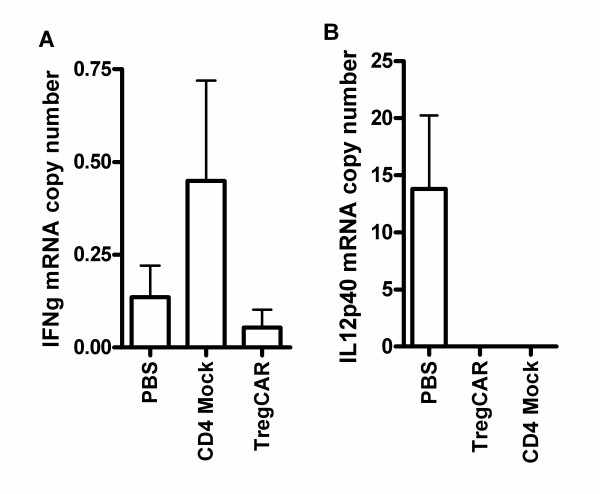
**Decreased expression of effector cytokines in CNS-targeting CAR Treg-treated brain.** Mice in three groups were given 1 × 10^5^ CAR Tregs, CD4^+^ Mock T cells or PBS alone by i.n. administration at the peak of EAE inflammation (15 days post-EAE immunization). Fifteen days post cell treatment, brain biopsies from five EAE mice per group (CAR Tregs, Mock CD4^+^ T cells and PBS) were analyzed for expression of effector cytokines (IL-12 and IFNγ) by quantitative RT-PCR. Error bars represent standard error of the mean (SEM).

In addition to the noted markers of recovery and myelination, the levels of Th1-associated cytokines were measured by quantitative PCR analysis of tissues from the same brains. These results revealed lower levels of the T cell associated IFNγ mRNA in mice treated with CAR Tregs compared to control brain tissue such as mice treated with PBS or Mock-transduced cells (Figure 6). IL-12, on the other hand, is only detected in PBS mice demonstrating that DC maturation may be compromised in both CAR Treg and Mock groups.

## Conclusions

Tregs are developed in the thymus (natural Tregs) or in the periphery in response to cytokines such as TGFβ. In autoimmunity, the patients may have genetic variations leading to reduced, or nonfunctional, Tregs. They may, as well, have deficits in other signalling pathways that affect the Treg suppression mechanisms. Severe systemic autoimmunity, such as immune polyendocrinopathy enteropathy X-linked syndrome (IPEX) shows *FoxP3* mutations that block the Treg differentiation. It is difficult to dissect if natural or peripherally derived Tregs are the most important for controlling autoimmunity, but since the natural Tregs commonly have affinity for self-antigens these Tregs likely have a predominant role in blocking emerging autoimmunity [2,3,5]. In the current investigation, CD4^+^ T cells were modified utilizing a lentiviral vector system to express a chimeric antigen receptor (CAR) targeting myelin oligodendrocyte glycoprotein (MOG) *in trans* with the murine *FoxP3* gene that drives Treg differentiation. In that sense, the gene-engineered Tregs are peripherally derived cells. However, they express FoxP3 and have a strong affinity to a self-antigen via the CAR receptor, so they are also similar to natural Tregs. The genetically engineered Tregs demonstrated suppressive capacity *in vitro* and reduced disease symptoms in mice with active EAE *in vivo*. The suppressive effects of Tregs in murine models of autoimmune pathology have provoked an interest in clinical translation. Transfer of Tregs into animals with autoimmunity provides protection but, to date, there are no records of clinical trials using adoptive transfer of Tregs in humans with autoimmune diseases. Tregs have, however, been used in the clinic to induce transplantation tolerance [21]. One problem with Tregs is sorting and expanding the population since FoxP3, the most reliable marker, is only present intranuclearly. It is also a major challenge to produce antigen-specific Tregs for cell therapy since that would require antigen-stimulation for an extended time. However, Tregs can be generated from naive CD4^+^ T cells by gene transfer of *FoxP3*[22]. Using retroviral gene transfer of murine *FoxP3* into CD4^+^CD25^-^ T cells, Chai and coworkers generated Tregs and used them in an animal model for transplantation with promising results [23], and this was later confirmed using a lentiviral vector system [24].

Genetically engineered and cultured Tregs have been evaluated for treatment efficacy in a wide variety of adoptive transfer models of autoimmune diseases [9,23,25-27]. However, systemically delivered Tregs may result in recipient failure to respond to infectious disease, or they may not accumulate in sufficient amounts at the correct location. Mekala and co-workers redirected murine CD4^+^CD25^+^ Tregs by using a chimeric antigen-MHC/ζ receptor targeting myelin basic protein (MBP) in the EAE model. In their study MBP-specific Tregs were able to suppress EAE-inflammation [28]. Hombach and coworkers have redirected human CD4^+^CD25^+^ Tregs by using retroviral transfer of a recombinant anti-CEA-immunoreceptor to target the inflamed intestine, with promising results [29]. In the present study, we have used chimeric antigen T cell receptors, so-called CARs, to direct the T cells to MOG present in the CNS [17]. The construct also contained murine FoxP3 to drive the transduced CD4^+^ T cells toward a Treg stable phenotype.

The engineered Tregs expressed both CAR and FoxP3, and in assays testing their function they significantly decreased T cell proliferation even in the presence of LPS-stimulated macrophages that are thought to take part in the transformation of Tregs into Th17 effector cells due to their production of activating cytokines [30,31]. Binding to MOG^+^ cells by their CAR did not change their suppressive function either, as indicated by co-culturing CAR Tregs, MOG^+^ cells and stimulated T cells. Activated macrophages are part of the pathology of MS and the CAR T cells were challenged with such cells in the suppressive assays to exclude that they lose their function in the presence of macrophages. There was a somewhat decreased suppressive capacity noted in these groups, but it did not reach significance.

GFP-expressing CAR Tregs were then used to track the immunofluorescence in the brain 24 hours after an i.n. cell administration. Analysis of GFP-positive immunofluorescence in the brains of naïve mice revealed clusters of fluorescent cells in various brain areas. Immunofluorescence was localized, for instance, in the olfactory bulb, orbital and ectorhinal cortex, but also in the Purkinje cells and white matter of the cerebellum. The selective GFP immunofluorescence of the Purkinje cells and other cells that are not matching was detected in the cerebellum. The selective GPF immunofluorescence of the Purkinje cells, and/or other cells that are not matching the features of Tregs, may possibly be related to a vesicular transfer and uptake of the GFP protein. Further investigations are needed to establish that the observed immunofluorescence is due to GFP-expressing CAR Tregs, fusion with other cells or cell debris that has been taken up by other cells.

A previous study has described that myelin-specific Tregs accumulate in the CNS but fail to control autoimmune inflammation [32]. This depended on the resistance of local effector T cells to suppression, partly due to IL-6 and TNF production. However, McGeachy and coworkers described that transfer of low numbers of CD4^+^CD25^+^ cells from the CNS of recovering mice before EAE reinduction reduces disease severity in recipients [33], demonstrating the potential of Treg therapy in EAE. Our present results clearly demonstrated that Tregs (engineered or not) could reduce disease symptoms in mice with active EAE upon both i.n. and i.p. delivery. Hence the effector cell resistance to Treg suppression demonstrated by Korn and coworkers [34] may not occur using engineered Tregs. However, the mock-transduced CD4^+^ T cells containing a mixture of natural FoxP3^+^ Tregs and naïve T cells did not completely cure EAE. When delivered i.n. mice treated with CD4^+^ Mock T cells could recover from EAE nearly as well as with CNS-targeted CAR Tregs, but the effect was not optimal, since only CAR Tregs could generate mice resistant to an additional EAE challenge 30 days post-treatment. If delivered i.p. the difference between targeted and nontargeted Tregs became more evident.

Immunohistochemical evaluation of recovery (GFAP) and myelination (MBP) of axons in the brain confirmed recovery and revealed decreased damage to axons in mouse brains treated with CAR Tregs compared to control groups. Furthermore, mice treated with CAR Tregs had reduced levels of effector cytokines (IL-12 and IFNγ) in brain tissue compared to both mice treated with crude T cells and PBS, thus indicating the different qualities of the targeted and non-targeted Tregs in suppressing inflammation.

A problem with adoptive transfer of Tregs is the inadequate number of cells reaching the target. Cell numbers decrease during migration and the risk of therapeutic cells ending up in vital and/or reproductive organs must be taken into consideration. The olfactory pathways have been extensively investigated as potential entry for pharmaceutical drugs into the brain [35,36]. Recently, i.n delivery has been examined as a potential route of administration for transplantation of cells into the brain with the advantage of reducing cell doses required for therapeutic efficacy while, at the same time, decreasing systemic exposure [32,37,38]. In this study, we further demonstrated intranasal administration of engineered GFP + Treg cells carrying a MOG-targeting receptor can be delivered via the nostrils and access the brain.

A migration of engineered Treg in the olfactory pathways to the brain may occur via extracellular channels comprising olfactory ensheathing cells surrounding the olfactory neurons or via perivascular spaces from the nose to the brain. In addition, a migration into the general blood circulation cannot be excluded since the nasal mucosa is highly vascularized. A previous report demonstrated migration of cells from the nasal mucosa through the cribriform plate along the olfactory neural pathway into the brain and cerebrospinal fluid (CSF) following i.n. cell administration [32]. In that study, only a low number of cells were tracked up to one hour post-instillation. In the present study, we observed clusters of immunofluorescent cells in the brain 24 hours post-instillation in naïve mice. Because EAE mice treated once with CAR Tregs were still protected against an additional EAE rechallenge the cells may still be intact >30 days post initial cell therapy, but other explanations are possible. Extrapolating to the human situation, the concept presented in the current paper could facilitate administration of therapeutic cells without compromising the patient’s safety or the number of cells reaching its target. In summary, i.n. cell administration of CNS-targeted CAR Tregs can be delivered to the brain using i.n. cell administration and efficiently hamper the disease symptoms as well as protect against an additional EAE challenge. The novel findings in this report warrant clinical translation.

## Competing interests

The authors declare that they have no competing interests with the contents in this paper. However, Dr. Loskog is the CEO of Lokon Pharma AB, an advisor of NEXTTOBE AB, and has a royalty agreement with Alligator Bioscience AB.

## Authors’ contributions

MF, EP, EB, and AL designed research and wrote the paper. MF, EP, JB, RH performed experiments. BN, ME, BL, RH and AL designed separate protocols and provided the necessary scientific input to execute the project. All authors read and approved the final manuscript.
